# Rapid 65-min SYBR-Green PCR Assay for Carbapenem Resistant *Klebsiella* and *Acinetobacter* Detection

**DOI:** 10.3390/microorganisms13112590

**Published:** 2025-11-13

**Authors:** Sebnem Bukavaz, Kultural Gungor, Hakan Kunduracılar, Zerrin Yulugkural

**Affiliations:** 1 Health Services Vocational College, Trakya University, 22030 Edirne, Turkey; 2 Department of Infectious Diseases and Clinical Microbiology, Kırklareli University, 39100 Kırklareli, Turkey; 3 Department of Infectious Disease and Clinical Microbiology, Trakya University, 22030 Edirne, Turkey

**Keywords:** SYBR-Green PCR, *Klebsiella pneumoniae*, *Acinetobacter baumannii*

## Abstract

This study developed a rapid and reliable SYBR-Green semiplex PCR assay for simulta-neous detection of major carbapenem resistance genes in *Klebsiella pneumoniae* and *Acinetobacter baumannii*. Two primer sets were used: one to detect *bla*KPC, *bla*NDM-1, and *bla*OXA-48 genes in *K. pneumoniae* and *bla*OXA-23 in *A. baumannii*, and another to amplify conserved 16S rRNA gene regions as internal controls. The intra- and inter-assay coeffi-cient of variation ranged from 0.03% to 3.8%. Standard curves exhibited excellent linearity across six logarithmic scales (10^1^–10^6^ DNA copies/µL), with detection limits of 10–10^2^ DNA copies/mL. Melting temperatures (Tm) were: 88.85 °C (KPIC), 90.65 °C (NDM-1), 89.45 °C (KPC), 84.23 °C (OXA-48), 87.81 °C (OXA-23), and 80.67 °C (ABIC). The SYBR-Green Semiplex PCR assay offers a rapid (65 min turnaround), cost-effective, and sensitive method for early detection of carbapenem-resistant pathogens, enabling timely targeted therapy and improved infection control by potentially reducing empirical antibiotic use before culture confirmation.

## 1. Introduction

Healthcare-associated infections (HCAIs) refer to infections that develop in patients while they are receiving medical treatment or care [1]. HCAIs are typically defined as infections that develop ≥48 h after hospital admission or within 30 days following exposure to healthcare services or interventions [2]. They contribute to the increasing problem of antimicrobial resistance. HAIs are the most problematic infections in terms of mortality, morbidity, and financial expenditures [3]. A recent study showed that the overall device-associated HAI rate was 1.88% in hospitals and 4.19% in intensive-care units (ranging from 1.22 to 28.42) [4]. The prevalence of HAI reported by hospitals in the Eastern Mediterranean and Southeast Asia is 11.8% and 10.0%, respectively [5]. Information on HAIs is not updated annually, but new reports on HAI infections and their distribution among the different types of infections for each country are published every 3–4 years by Public Health Concerns. According to the Surveillance of Antimicrobial Resistance in Europe (EARS-Net) 2022 report, the critical bacteria in terms of antibiotic resistance are categorized as *Enterococcus faecium*, *Staphylococcus aureus*, *Klebsiella pneumoniae*, *Acinetobacter baumannii*, *Pseudomonas aeruginosa*, and *Enterobacter* spp. (ESKAPE), and their current resistance patterns are reported [6]. The 2024 national healthcare-associated infection surveillance report indicates carbapenem resistance rates among HAI of 92.18% in *A. baumannii*, 66.56% in *K. pneumonia* and 67.60 in *P. aeruginosa* [7]. These bacteria are transmitted through the fecal–oral route as a result of inadequate hygiene practices and colonization of the intestine, particularly in animals and humans. Close contact is frequently the primary cause of contamination and the spread of resistant colonies [8]. Travel increases the risk of the global spread of carbapenem resistance or multidrug-resistant bacteria, which healthcare providers should consider when treating patients [9]. Surveillance of healthcare-associated infections, including resistance rates and enzyme patterns, in hospitals is crucial for infection prevention and the appropriate use of antibiotics [10]. Furthermore, integrating this surveillance with diagnostic algorithms that incorporate rapid PCR testing can facilitate pre-emptive isolation and effective outbreak control, thereby strengthening the rationale for the assay.

Molecular PCR methods offer faster results than traditional cultures, improving hospital infection control and preventing delays and facilitate early treatment and targeted antibiotic selection through rapid, specific detection of hospital infection agents [11,12]. Multiplex and semiplex (semi-multiplex) PCR types are preferred for diagnosing multi-resistant bacteria in intensive care units because they simultaneously detect multiple resistance genes or pathogens [13]. Simplex, Semiplex and Multiplex PCR types are simply compared at Table 1 [14,15].

Recent advances in molecular diagnostics for hospital-acquired infection agents and antimicrobial resistance genes have enabled very low limits of detection (LoDs). For example, multiplex real-time PCR assays for major carbapenemase genes (VIM, IMP, NDM, KPC, OXA-48) have achieved LODs as low as 2–15 CFU/reaction (VIM) and 4–42 CFU/reaction (KPC) in optimized conditions [16].

The aim of this study is to develop a molecular Semiplex SYBR-Green PCR assay for the rapid detection of carbapenem resistance. The method is designed in this study to be accurate, sensitive, economical, and repeatable, capable of directly detecting three of the most common target genes *bla*NDM-1, *bla*KPC, and *bla*OXA-48 of *K. pneumoniae* as well as the addition of *bla*OXA-23 for *A. baumannii* from only reference strains after quick DNA extraction.

## 2. Materials and Methods

### 2.1. Bacterial Strains and Cultivation

In this study, six well-characterized bacterial reference strains were used: *K. pneumoniae* BAA-1705 (kpc+), *K. pneumoniae* derived 2524 (oxa 48+), *K. pneumoniae* BAA-1706 (kpc–control), *K. pneumoniae* ATCC BAA-2473 (ndm-1+), *Acinetobacter baumannii* derived from NCTC 13304 (Oxa-23+), and *A. baumannii* ATCC 19606 (wild-type). All strains were cultured onto %5 blood-sheep agar plates (Oxoid, Basingstoke, UK) and incubated for 8 h at 37 °C under aerobic conditions, following the manufacturer’s recommendations.

### 2.2. DNA Extraction and Dilution of DNA for Quantification

Genomic DNA was extracted via a modified boiling lysis method [17]. Briefly, fresh bacterial colonies were suspended in 200 µL nuclease-free water, adjusted to a 0.5 McFarland standard, and incubated at 100 °C for 10 min. After centrifugation at 12,000× *g* for 5 min, 100 µL of the supernatant containing DNA was collected and stored at −20 °C. A NanoDrop 2000 spectrophotometer (Thermo Fisher Scientific, Waltham, MA, USA) was used to determine DNA concentration and purity.

For quantitative SYBR-Green PCR, DNA standards from each reference strain (KPC, NDM, OXA48, OXA-23) were prepared at an initial concentration of 3.2 × 10^7^ CFU/mL and serial diluted to 10^2^ CFU/mL. Each dilution was tested in 5–7 replicates to construct standard curves.

### 2.3. Primer Design

Primers were designed using Primer3 v.4.1.0 software and validated with NCBI Primer-BLASTtool v.1 to ensure specificity against bacterial genomes. Details of the primers, including sequences, expected amplicon sizes, and references, are provided in Table 2.

### 2.4. Optimization of Semiplex SYBR-Green Real Time PCR

The Semiplex SYBR-Green PCR panel (SBR-KPAB) was developed to detect major carbapenemase genes (KPC, NDM, OXA-48, OXA-23), together with internal controls specific for *K. pneumoniae* (KP-IC) and *A. baumannii* (ABIC). Escherichia coli 16S rRNA was included as a negative control to exclude cross-reactivity. Unlike classical multiplex PCR, the semiplex design separates gene panels to improve specificity and reduce primer-dimer formation. Due to the lower amplification efficiency and higher risk of nonspecific amplification observed in overlong amplicons, each target was analyzed in separate wells rather than multiplexed in a single reaction.

PCR was performed in 20 µL reactions using 1× SYBR-Green Master Mix (Atlas Biotechnologies, Ankara, Turkey), 0.2 µM primer set, 3 µL DNA template, and nuclease-free water. Amplification was performed on an Applied Biosystems™ StepOne™ Real-Time PCR System (Thermo Fisher Scientific) with initial denaturation at 95 °C for 10 min, followed by 40 cycles of 95 °C for 15 s and 60 °C for 60 s.

Melting curve analysis followed amplification: 95 °C for 15 s, 60 °C for 1 min, and a gradual increase to 95 °C at 0.1 °C/s. All assays were performed in triplicate for intra- and inter-assay validation.

### 2.5. Data Analysis

Amplification data were analyzed using StepOne™ software v2.3 (Thermo Fisher Scientific, USA). The positivity threshold was defined as a cycle threshold (Ct) ≤ 35, based on control strain performance. Carbapenemase genes were confirmed by both specific melting curve profiles and the presence of characteristic Ct values.

### 2.6. Statistical Analysis

Standard curves were generated for each gene target, and linear regression analysis was used to calculate amplification efficiency (E) and correlation coefficients (R^2^). Reproducibility was assessed by calculating intra- and inter-assay coefficients of variation (CV%), with CV values below 5% considered acceptable [18].

The SYBR-Green-based PCR assay’s analytical performance was evaluated for reproducibility, precision, and sensitivity. The cycle threshold (Ct), defined as the PCR cycle at which fluorescence intensity exceeded the baseline, marked the first detectable target DNA amplification. Reactions were performed in triplicate, and only results with a Ct standard deviation (SD) ≤ 0.5 among replicates were accepted to ensure accuracy and repeatability. Intra- and inter-assay variability were determined using the coefficient of variation (CV) of Ct values, calculated as: CV (%) = SD/Mean Ct × 100

Intra-assay and inter-assay coefficients of variation (CVs) were calculated from replicate reactions within the same run and from runs on different days, respectively. CVs < 5% (intra-assay) and <10% (inter-assay) indicated high precision.

The limit of detection (LoD_95_%) was determined by testing serial dilutions of DNA standards (10^0^–10^5^ copies/reaction) in at least eight replicates. LoD_95_% was defined as the lowest concentration with ≥95% positive amplification, as determined by probit regression analysis of detection probability versus target copy number.

All statistical analyses were performed using Microsoft Excel (Microsoft Corporation, Redmond, WA, USA).

## 3. Results

### 3.1. Validation of Primer Specification

This study employed specifically designed primers targeting individual bacterial genes. Standardized semiplex SYBR-Green PCR conditions were used for all selected genes and housekeeping genes serving as internal controls for *K. pneumoniae* (KPIC) and *A. baumannii* (ABIC).

### 3.2. DNA Concentrations for Quantitative Semiplex SYBR-PCR

SYBR-Green PCR was performed using the primer manufacturer’s recommended annealing temperatures and amplification cycles. DNA dilutions (10^7^ to 10^2^) were prepared using 565 ng/µL (NDM-1), 780 ng/µL (KPC), 655 ng/µL (OXA-48), and 875 ng/µL (OXA-23) primer concentrations (Table 3). Linearity and limit of detection were evaluated using serial dilutions of reference carbapenemase genes. All R^2^ values exceeded 0.974, and amplification efficiencies were above 94% for the Semiplex SYBR-Green PCR.

Prepare serial 10-fold dilutions with 100 µL final volume per tube. To obtain a 10^7^ copies/µL stock (100 µL final) from a ~1 × 10^9^ copies/µL working stock:

Volume of working stock = (C_target × V_total)/C_working = (1 × 10^7^ copies/µL × 100 µL)/1 × 10^9^ copies/µL = 1 µL working stock + 99 µL diluent.

Perform serial 10-fold dilutions as follows:

10^7^ → 10^6^: Transfer 10 µL of the 10^7^ dilution into 90 µL of diluent (mix well) to yield 100 µL of 10^6^.

Repeat the 10 µL → 90 µL transfers to generate 10^5^, 10^4^, 10^3^, and 10^2^ dilutions.

Technical replicates: triplicate wells (*n* = 3) per dilution point per run.

### 3.3. Melting-Curve Analysis

Melting-curve analysis of positive reference strains yielded distinct melting peaks for KPIC, NDM-1, KPC, OXA-48, OXA-23, and ABIC primers at 88.85 ± 0.11 °C, 90.65 ± 0.20 °C, 89.45 ± 0.30 °C, 84.23 ± 0.11 °C, 87.81 ± 0.11 °C, and 80.67 ± 0.30 °C, respectively. Amplicons of KPC (498 bp), NDM-1 (621 bp), OXA-48 (774 bp), and OXA-23 (452 bp) exhibited distinct melting behaviors, with temperature differences of 1.06 °C, 1.2 °C, 6.56 °C, and 3.58 °C, respectively, at a melting rate of 0.1 °C/s. The observed similarities between KPC and NDM-1 melting curves, attributed to fluorescence signal loss influenced by melting rate and primer concentration, were further investigated by repeating SYBR-PCR with a 10-fold lower primer concentration. This resulted in a melting point shift from 88.7 °C to 83.2 °C. To address this limitation, the SBR-KPAB PCR panel was modified to include a separate internal control well, allowing for verification of melting peaks even with similarities.

### 3.4. Reproducibility of the SBR-KPAB PCR

The coefficients of variation (%) for intra- and inter-assay reproducibility were 0.06–3.7 (KPC), 0.95–3.87 (NDM-1), 0.28–1.45 (OXA-48), and 0.02–0.17 (OXA-23), respectively (Table 3).

**Figure 1:**
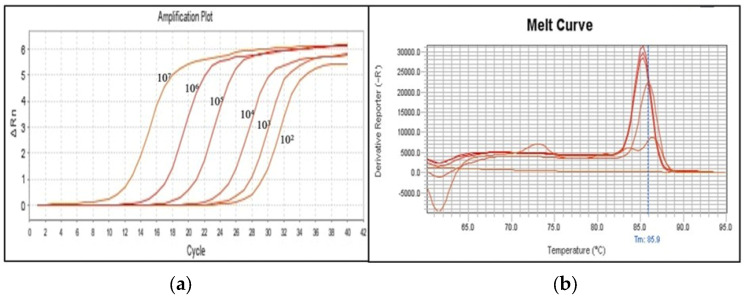
Amplification plot (**a**) and melting-curve analysis (**b**) of an unidentified carbapenem-resistant strain. The OXA-48 assay produced a clear positive signal, showing a distinct melting peak at approximately 85 °C. For this analysis, 67 ng/µL DNA extracted from an unidentified carbapenem-resistant reference strain was serially diluted (1:10) from 10^7^ to 10^2^ and tested by individual SYBR-Green PCR assays using KPIC, KPC, NDM-1, OXA-48, 23, and ABIC primer sets.

The SBR-KPAB quantitative PCR assay was validated by isolating and quantifying DNA from an unidentified carbapenem-resistant strain using a Nanodrop spectrophotometer. Serial dilutions of the DNA (10^7^ to 10^2^) were subjected to SYBR-PCR, which detected NDM-1 (Table 4) Melting curve analysis confirmed the reliability of the result (Figure 1). The Semiplex SYBR-Green PCR assay demonstrated a detection limit of 1–3 copies/mL based on DNA concentration, with PCR efficiency ranging from 94% to 100% across reference strains.

## 4. Discussion

Rapid and accurate detection of carbapenemase genes is critical for timely therapeutic decisions, as conventional culture-based methods are often too slow to provide comprehensive resistance data [19]. Currently, phenotypic tests, MALDI-TOF MS, and probe-based PCR methods are widely used for the detection of hospital-acquired infections and associated resistance enzymes [20,21]. Among these, probe-dependent PCR offers enhanced sensitivity and specificity due to the use of labeled probes, enabling detection and quantification of targets at very low copy numbers, including viral genomes [22]. Although SYBR-Green real-time PCR assays are limited by non-specific primer binding, which can compromise sensitivity and reliability, this limitation was addressed in our study by approximating fluorescence loss to allow more reliable detection of weak melting peaks influenced by melting rate and primer concentration [23]. Optimization of PCR conditions, particularly primer specificity and annealing temperature, was performed using NCBI Primer-BLAST data to ensure unique amplification for each target. With this optimization, the Semiplex SYBR-Green PCR assay was able to simultaneously detect carbapenemase genes a similar detection limit, with concentrations of 1, 1.4, and 2.3 copy/mL for KPC, NDM-1, and OXA-48,23, respectively, and identify bacterial species within approximately 65 min. The LoD_95_ value was calculated for each dilution, taking into account the minimum dilution amount. Based on the PCR reaction results obtained from the 10^2^–10^7^ serial dilutions, the LoD_95_ calculation was performed and values indicated at 94–100% positivity were found. This rapid turnaround is particularly advantageous for patients with hospital-acquired infections (HCAIs), where prolonged hospitalization increases.

The rapid and accurate detection of carbapenemase genes in clinical microbiology laboratories is essential for guiding therapeutic decisions and ensuring appropriate antibiotic selection. Conventional culture and antibiogram techniques are time-consuming and often fail to provide comprehensive resistance data [19]. Currently, phenotypic tests, MALDI-TOF MS, and probe-based PCR methods are widely used for the detection of hospital-acquired infections and associated resistance enzymes [20,21]. Among these, probe-dependent PCR offers enhanced sensitivity and specificity due to the use of labeled probes, enabling detection and quantification of targets at very low copy numbers, including viral genomes [22]. The emergence of antibiotic resistance is a multifactorial issue, driven by the misuse of antibiotics, inadequate infection control practices, and inappropriate dosing [24]. Infections caused by carbapenem-resistant Enterobacteriaceae are associated with high mortality rates, ranging from 26% to 50% globally, with even higher rates in pediatric patients [25]. HCAIs such as ventilator-associated pneumonia (VAP) remain a leading cause of mortality, particularly in intensive care units, where carbapenem-resistant Gram-negative bacteria are frequently implicated [26]. The economic burden of these infections is considerable, including the costs of prolonged antibiotic therapy, extended hospital stays, patient isolation measures, and loss of workforce productivity [27]. In some countries, such as Turkey, reimbursement mechanisms to cover these additional costs are lacking, underscoring the need for improved infection control strategies. In contrast, France has introduced compensation systems for patients with hospital-acquired infections not attributable to their own microbiota, an approach that may encourage better infection control practices and reduce the economic impact of antibiotic resistance [28].

Transmission of resistant bacteria, including *E. coli*, *A. baumannii*, and *K. pneumoniae*, is facilitated by poor hygiene, close contact, and fecal–oral contamination [29]. Horizontal gene transfer, frequently mediated by insertion sequences (ISs), accelerates the spread of resistance determinants, while the hospital environment serves as a reservoir for epidemic strains and supports ongoing outbreaks [30]. Rapid diagnostic methods, such as the Semiplex SYBR-Green PCR assay developed in this study, provide results within approximately one hour and hold promise for direct application to clinical samples. This speed and accuracy can play a vital role in both patient management and infection control. This speed and accuracy can play a vital role in both patient management and infection control, contributing to early identification, isolation, and effective outbreak control.

Although there is no universally accepted “gold standard” treatment for carbapenem-resistant infections, combination therapies involving two or more antibiotics have shown improved clinical outcomes. Ceftazidime-avibactam (CAZ-AVI), for instance, has demonstrated efficacy against certain carbapenem-resistant strains, although its effectiveness varies across carbapenemase types [31,32]. Therefore, precise and rapid discrimination of carbapenemase enzymes using reliable diagnostic assays is crucial for guiding targeted therapy and improving patient prognosis.

## 5. Conclusions

In summary, we developed a novel SYBR-Green-based semiplex real-time PCR assay capable of simultaneously detecting bacterial species and carbapenemase genes in reference strains. This assay has the potential to provide a rapid, cost-effective, and accurate tool for monitoring transmissible carbapenem resistance and assessing the risk of infection outbreaks.

Limitations: The study’s limitation is that it used only reference strains. Experimental specificity testing and clinical validation of the semiplex SYBR-Green assay were not performed in the current study. The analytical performance reported here (sensitivity, LoD, and intra-/inter-assay precision) was determined using purified nucleic acid and contrived samples; therefore, matrix effects and potential cross-reactivity with non-target organisms remain to be assessed. Future work will include a comprehensive cross-reactivity panel and prospective clinical evaluation to determine diagnostic accuracy (sensitivity, specificity, Positive and Negative Predictive Value) in real clinical specimens.

## Figures and Tables

**Table 1:** Comparison of Simplex, Semiplex and Multiplex PCR.

Feature	Simplex PCR	Semiplex PCR	Multiplex PCR
Number of Targets	1	2–3 (limited number)	Multiple (3 or more, often many)
Primer Pairs Used	1 pair	2–3 pairs	Multiple primer pairs
Optimization Difficulty	Low	Moderate	High (due to primer interactions)
Efficiency/Compatibility	Low (one target at a time)	Moderate	High (simultaneous detection)
Application	Single gene/pathogen	Limited panel of genes/pathogens	Broad panels, comprehensive screening
Specificity and ΔTm Separation	High; no cross-reactivity	Moderate; ΔTm > 3 °C recommended	Requires strict ΔTm control (>5 °C) to avoid overlap
Risk of Dimers/Artefacts	Minimal	Possible between some primers	High; primer–dimer and mispriming frequent
Cost/Time	Higher cost and time per target	Moderate (fewer reactions)	Cost-effective for large panels but time-consuming optimization
Equipment Requirements	Standard thermal cycler	Real-time PCR with multichannel detection preferred	Advanced real-time PCR or digital systems with precise temperature control
Application	Single gene or pathogen detection	Limited panel (e.g., resistance genes)	Broad panels, comprehensive diagnostics or surveillance

**Table 2:** Primers used in the current study.

Primer	Sequence (5′-3′)	Product Size (bp)	Length (bp)	Tm (°C)	%GC	Max Self-Compl. (nt)	Self-Compl. Risk	Dimer/Hairpin Penalty
*bla*KPC-2_F	CGGAACCATTCGCTAAACTC	498	20	51.3	50.0	3	Low	Low
*bla*KPC-2_R	AGCCCAGTGTCAGTTTTTGT		20	51.98	45.0	2	Low	Low
*bla*NDM-1_F	GGTTTGGCGATCTGGTTTTC	621	20	51.97	50.0	4	Moderate	Moderate
*bla*NDM-1_R	CGGAATGGCTCATCACGATC		20	53.2	55.0	4	Moderate	Moderate
*bla*OXA-48_F	GAGCACTTCTTTTGTGATGGCTTG	774	24	55.26	45.83	3	Low	Low
*bla*OXA-48_R	ATGCGTGTATTAGCCTTATCGGC		23	55.33	47.83	3	Low	Low
*bla*OXA-23_F	AATACAGAATATGTGCCA	452	18	42.16	33.33	4	Moderate	Moderate
*bla*OXA-23_R	TCCATTGCCCAACCAGTCTTTCC		23	57.19	52.17	3	Low	Low
*K.pneumoniae*_F	CCACACTGGAACTGAGACAC	298	20	52.4	55.0	3	Low	Low
*K.pneumoniae*_R	TCACATCCGACTTGACAGAC		20	51.55	50.0	3	Low	Low
*A.baumannii*_F	GGGAGCAAACAGGATTAGATAC	311	22	50.48	45.45	2	Low	Low
*A.baumannii*_R	ACCCAACATCTCACGACAC		19	51.68	52.63	2	Low	Low

**Table 3:** Reproducibility of the Semiplex SYBR Real-Time PCR-assay.

DNA Concentrations (ng/mL)	Ct Mean	St. Deviation	CV %	DNA Concentrations (ng/mL)	Ct Mean	St. Deviation	CV %
**KPC**				**NDM-1**			
**780**	13.61	0.5	3.71	565	17.95	0.69	3.87
**78**	16.82	0.4	2.5	5.65	22.27	0.48	2.15
**7.8**	19.85	0.05	2.5	5.65	24.85	0.23	0.95
**0.78**	22.32	0.01	0.3	0.565	27.62	0.41	1.49
**0.078**	25.41	0.4	0.06	0.0565	29.72	0.38	1.29
**0.0078**	26.88	0.11	1.6	0.00565	30.07	0.75	2.50
**0.00078**	30.6	0.11	0.4	0.000565	29.94	0.59	1.98
**OXA-48**				**OXA-23**			
**655**	16.94	0.2	1.45	875	11.88	0.02	0.17
**65.5**	19.81	0.05	0.28	87.5	13.93	0.01	0.1
**6.65**	23.07	0.5	2.48	8.75	15.23	0.04	0.3
**0.665**	26.21	0.6	2.56	0.875	17.59	0.01	0.05
**0.0655**	27.57	0.6	2.32	0.0875	20.11	0.02	0.08
**0.00655**	29.35	0.4	1.57	0.00875	24.15	0.02	0.06
**0.000655**	30.68	0.2	0.70	0.000875	28.42	0.01	0.02

**Table 4:** Serial Dilution of 67 ng/µL.

Step	Dilution	Volume of Previous Solution	Volume of Diluent	Resulting Concentration
1	1:10^3^	1 µL stock	999 µL buffer	7.9 × 10^10^ copies/mL
2	1:10^3^	1 µL step1	999 µL buffer	7.9 × 10^7^ copies/mL
3	1:10^3^	1 µL step2	999 µL buffer	7.9 × 10^4^ copies/mL
4	1:100	10 µL step3	990 µL buffer	7.9 × 10^2^ copies/mL
5	1:10	100 µL step4	900 µL buffer	79 copies/mL (≈10^2^)
6	1:10	100 µL step5	900 µL buffer	7.9 copies/mL (≈10)

## Data Availability

The original contributions presented in this study are included in the article. Further inquiries can be directed to the corresponding author.
